# Prevalence and associated factors of metabolic syndrome among Omani adults with mental illnesses: a cross-sectional study

**DOI:** 10.1038/s41598-025-25948-8

**Published:** 2025-11-25

**Authors:** Faisal Al Rashdi, Omar Al Omari, Blessy Prabha Valsaraj, Nasser Al-Sibani, Aziza Al Sawafi, Iman Al Hashmi, Maen Aljezawi, Mohammad Al Qadire, Mohammed ALBashtawy, Fadwa Alhalaiqa, Nasser AL Salmi

**Affiliations:** 1 College of Nursing, Sultan Qaboos University, Muscat, Oman; 2 College of Medicine and Health Sciences, Sultan Qaboos University, Muscat, Oman; 3 Faculty of Nursing, Al al-Bayt University, Mafraq, Jordan; 4 Clinical Affairs, College of Nursing, QU Health, Qatar University, Doha, Qatar

**Keywords:** Associated factors, Mental illnesses, Metabolic syndrome, Prevalence, Diseases, Endocrinology, Health care, Medical research, Risk factors

## Abstract

Individuals with mental illnesses are at increased risk of developing metabolic syndrome (MetS), a cluster of cardiovascular and metabolic abnormalities. Despite growing evidence globally, there is a lack of research in the Gulf region, particularly in Oman. This study is the first to assess the prevalence and associated factors of MetS among Omani adults with mental illnesses. A descriptive cross-sectional design was employed. Three diagnostic criteria were used to assess MetS: the International Diabetes Federation (IDF) criteria, the modified Adult Treatment Panel III (ATP III), and the Joint Statement of the IDF Task Force with other organizations, aiming to enhance comparability and diagnostic consistency. A convenience sample of 251 Omani adults with mental illnesses was recruited from the outpatient departments of two tertiary hospitals in Muscat, Sultanate of Oman. Metabolic syndrome prevalence was 29.9%, 30.3%, and 33.1% according to the ATP III-A, IDF, and Joint Statement criteria, respectively. Logistic regression analysis showed that greater waist circumference (OR = 1.06, 95% CI: 1.03–1.09, *p* < 0.001) and higher triglyceride levels (OR = 2.43, 95% CI: 1.40–4.22, *p* = 0.002) were significantly associated with increased odds of MetS. Higher physical activity (OR = 0.97, 95% CI: 0.95–0.99, *p* = 0.031) and the use of antidiabetic medication (OR = 0.17, 95% CI: 0.04–0.73, *p* = 0.017) were associated with lower odds. Our findings indicate substantial metabolic risk among Omani adults with mental illnesses. Accordingly, two concrete service actions are warranted: embed routine metabolic screening within psychiatric outpatient care; and implement structured lifestyle-support programs prioritizing physical-activity counselling and weight-management/nutrition referral pathways. These steps align directly with the study’s observed risk profile.

## Introduction

Mental illnesses are characterized by significant disruptions in a person’s thinking, emotional control, or actions^1^. The estimated number of people worldwide living with mental illnesses is approximately 792 million, representing 10.7% of the global population^2^. However, mental illnesses continue to be significantly under-reported compared to other disorders, particularly in lower-income countries^3^. Mental illnesses are also one of the main causes of disability worldwide^4^. Fiorillo and Sartorius^5^ reported that individuals with different mental illnesses have a reduced life expectancy of 10 to 25 years, with an increased risk of developing metabolic changes.

People living with mental illnesses face many challenges beyond the psychological symptoms, including a higher risk of developing physical health problems like Metabolic syndrome (MetS)^6^. Metabolic syndrome consists of various factors, including obesity, hypertension, dyslipidemia, and hyperglycemia, that increase the risk of cardiovascular disease, type 2 diabetes, and overall mortality^7^. Although MetS is common in the general population, its prevalence is greater among patients with mental illnesses^8^. This co-occurrence of physical and mental health disorders complicates the management of such conditions and contributes to the significant reduction in life expectancy seen in this population^9^.

Numerous studies have reported the bidirectional relationship between mental illnesses and MetS, linking this association to behavioral, pharmacological, and socioeconomic factors^10,11^. The use of certain psychotropic medications, particularly second-generation antipsychotics such as olanzapine and clozapine, has been associated with weight gain, dyslipidemia, and glucose dysregulation, thereby exacerbating the risk of MetS^12^. Additional contributing factors include higher body mass index (BMI), older age, lower educational level, and female gender^7^.

Lifestyle-related factors, such as poor dietary habits, low levels of physical activity, smoking, and poor cardiovascular fitness, have also been repeatedly linked to an increased risk of MetS in this population^8,13,14^. Furthermore, the nature of mental illness, its chronicity, and inconsistent care delivery further increase the risk^15^.

The high global incidence of MetS among patients with mental illnesses has become a significant public health concern^16^. In the Middle East and Gulf Cooperation Council (GCC) region, rising rates of both mental illness and metabolic disorders have been linked to rapid socioeconomic development, urbanization, and changes in dietary and lifestyle patterns. Recent reviews emphasize that the region faces unique sociocultural stressors, including conflict-related displacement, stigma surrounding mental health, and limited mental health infrastructure that complicate prevention and care efforts^17,18^. Despite this growing regional burden, few empirical studies have examined the coexistence of mental illness and metabolic syndrome in the Gulf countries, including Oman. The nutritional transition in Oman, characterized by increased consumption of processed foods high in saturated fats and sugars and a decline in traditional dietary patterns, may further contribute to the rising prevalence of MetS^19^.

Furthermore, cultural attitudes towards mental illness and a limited mental healthcare infrastructure present a challenge to the effective management of MetS patients with mental illnesses within this region, which may result in new study findings. Despite the rising burden of both mental illness and metabolic syndrome in Oman, no prior studies have explored their coexistence in this context. Therefore, this study aimed to provide the first Oman-based estimate of the prevalence of metabolic syndrome and to identify its associated factors among adults with mental illnesses.

## Methods

A descriptive cross-sectional design was used to examine the prevalence and associated factors of metabolic syndrome among adults with mental illnesses in Oman.

### Sample and settings

The sample comprised patients attending psychiatric outpatient departments at Al-Masarra Hospital and Sultan Qaboos University Hospital. These two leading government referral hospitals serve patients with a wide range of mental illnesses from all over Oman. The inclusion criteria were: (1) aged 18 years or older; (2) diagnosed with a mental illness for at least six months, (3) receiving psychotropic medication and being able to read and understand the Arabic language, and (4) able to provide consent .

The sample size was initially determined using a logistic-regression rule of thumb: n = (m × 10) + 50, where m is the anticipated number of predictors. With an estimated 20 predictors, the minimum required sample size was 250 participants. In addition, because one of the study’s primary aims was to assess the prevalence of MetS, we considered a precision-based criterion: assuming a prevalence of 30%, *n* = 250 yields a 95% confidence interval with an approximate half-width of ± 6% points, which we judged adequate for reporting the baseline prevalence. This combined justification supports the adequacy of the achieved sample for the study’s dual aims.

### Ethical approval

The study was approved by the Ministry of Health Research Committee (MoH/CSR/22/26640) and the Hospital Ethics Committee (EC/017/2023). The study adhered to the principles outlined in the Declaration of Helsinki. Every participant was treated with equal respect and dignity, and their privacy was respected. All necessary procedures were followed to safeguard participants’ privacy and confidentiality in accordance with legal requirements.

### Data collection

Data were collected between March and September 2023. Nurses at the participating psychiatric outpatient departments assisted the research team in identifying eligible participants. Individuals who met the inclusion criteria received a full explanation of the study and, if willing, provided written informed consent before completing a coded questionnaire.

Clinical data were also retrieved from the participant’s clinical records; however, it did not systematically capture antipsychotic exposure details needed for analysis, specifically, antipsychotic class (typical vs. second-generation), specific agent, daily dose, formulation (oral vs. long-acting injectable), duration of exposure, and adherence. As such, only the binary inclusion status “on psychotropic treatment” was uniformly available; detailed antipsychotic variables were not analyzable. Anthropometric measurements were obtained in a private area to maintain confidentiality.

## Measurement

### Outcome variable

#### MetS

MetS was assessed using three internationally recognized diagnostic criteria: the International Diabetes Federation (IDF) criteria, the modified Adult Treatment Panel III (ATP III), and the Joint Statement of the IDF Task Force with other organizations. All criteria emphasized central obesity, which was assessed by waist circumference as a key diagnostic element, using ethnic- and gender-specific cut-offs for Middle Eastern populations^20,21^. Waist circumference was measured at the midpoint between the 12th rib margin and the iliac crest during minimal respiration using a non-stretchable plastic measuring tape, following standardized procedures for all participants. The researchers employed different criteria to enhance sensitivity and comparability across international studies. Each diagnostic criterion is explained in Table 1.

**Table 1:** Diagnostic criteria for metabolic syndrome.

Component	IDF	Joint Statement	ATP III-A
Rule	Central obesity plus ≥ 2 of the following components	Any 3 of 5 components	Any 3 of 5 components
Waist circumference (cm)	≥ 94 (men); ≥80 (women)	≥ 94 (men); ≥80 (women)	> 102 (men); >88 (women)
Triglycerides (mmol/L)	≥ 1.7 or Rx	≥ 1.7 or Rx	≥ 1.7 or Rx
HDL (mmol/L)	Men: <1.0 or Rx; Women: <1.3 or Rx	Men: <1.0 or Rx; Women: <1.3 or Rx	Men: <1.0 or Rx; Women: <1.3 or Rx
Blood pressure (mmHg)	SBP ≥ 130 and/or DBP ≥ 85 or Rx	SBP ≥ 130 and/or DBP ≥ 85 or Rx	SBP ≥ 130 and/or DBP ≥ 85 or Rx
FPG (mg/dL [mmol/L])	≥ 100 (5.6) or type 2 DM or Rx	≥ 100 (5.6)	≥ 100 (5.6)

IDF—International Diabetes Federation; ATP III-A—Modified Adult Treatment Panel III; SBP/DBP—systolic/diastolic blood pressure; TG—triglycerides; HDL—high-density lipoprotein; FPG—fasting plasma glucose; Rx—on pharmacological treatment; DM—diabetes mellitus. Units: lipids in mmol/L; BP in mmHg; FPG in mg/dL (with mmol/L shown once in brackets).

### Independent variables

#### Demographics and clinical questionnaire

Age, gender, marital status, educational level, employment status, current smoking status, History of smoking, healthy diet, Hypertension, Diabetes mellitus, dyslipidemia, medication use, including hypertensive, antidiabetic, and statin medications. The Hypertension, Diabetes mellitus, and dyslipidemia diagnoses were retrieved from the patients’ records. Anthropometric measures included height, weight, and waist circumference.

#### Physical activity

Physical activity level was assessed using the Godin-Shephard Leisure-Time Physical Activity Questionnaire (GSLTPAQ), which categorized participants as “active,” “moderately active,” or “insufficiently active”. Scores were derived from strenuous, moderate, and mild exercises. GSLTPAQ is a validated and practical tool for classifying activity levels^22^; the Arabic version was validated for the study^23^.

### Statistical analysis

Data were entered and analyzed using IBM SPSS Statistics Version 23.0. Descriptive statistics using means and standard deviations were used to analyze continuous variables, while frequencies and percentages were used for categorical variables. Any variable in bivariate analyses has *p* < 0.05 identified as a potential correlate of metabolic syndrome. Variables meeting this threshold, including age, educational level, comorbidities, total number of medications, antihypertensive use, antidiabetic use, statin use, total number of chronic illnesses, Godin physical activity score, waist circumference, triglycerides level, and number of previous psychiatric admissions, were entered into a binary logistic regression model to identify independent predictors of MetS. The primary dependent variable for multivariable modeling was MetS defined by the Joint Statement criteria. Diagnostic components of MetS, including waist circumference and triglycerides, were also entered to quantify their relative contributions. Because these variables are integral to the MetS definition, their inclusion may introduce circularity. Therefore, associations for diagnostic components were interpreted cautiously, with primary emphasis placed on demographic and clinical characteristics, as well as physical activity, as correlates beyond the diagnostic criteria. Confounding was addressed by including all covariates in the final model. Multicollinearity was examined using VIF (< 2.0). Model fit was evaluated using the Hosmer–Lemeshow test, with α = 0.05 set as the significance level. Although participants were diagnosed with various mental illnesses, the conditions were analyzed as a single group due to the limited number of cases within each diagnostic category. Subgroup analyses were therefore not feasible without compromising statistical power or violating the assumptions of the regression model. We did not run separate multivariable models for each MetS definition because the prevalence estimates were similar across criteria; thus, a single harmonized outcome was used to improve parsimony and interpretability.

## Results

A total of 251 adults diagnosed with mental illnesses (average age 38.4, SD = 10.6, range = 18–70) participated in the study. Most participants were under 40 years old (56.6%), male (52.2%), married (50.6%), unemployed (51.4%), and had a secondary school education or lower (54.2%), and took medications independently (64.1%). The prevalence of current smoking was 10.8%, while 17.5% had a history of smoking. For more details, see Table 2.

**Table 2:** Sociodemographic and characteristics of the study participants (*n* = 251).

Characteristics	Category	*N*	%	M (SD)
Age				38.4 (10.6)
Total number of chronic illnesses				0.38 (0.67)
Godin Activity level				19.16 (18.92)
Waist circumference size				94.38 (15.58)
Triglyceride blood level (mmol/L)				1.27 (1.03)
*Number of psychiatric inpatient admissions				1.30 (2.20)
Gender				
	Male	131	52.2	
	Female	120	47.8	
Marital status				
	Single	124	49.4	
	Married	127	50.6	
Educational level				
	Secondary school or less	136	54.2	
	Above secondary school	115	45.8	
Employment Status				
	Employed	85	33.9	
	Unemployed	129	51.4	
	Retired	37	14.7	
Hypertension (HTN)				
	Yes	28	11.2	
	No	223	88.8	
Diabetes mellitus (DM)				
	Yes	34	13.5	
	No	217	86.5	
Dyslipidemia				
	Yes	13	5.2	
	No	238	94.8	
Any medication				
	Yes	71	28.3	
	No	180	71.7	
Caregiver at home				
	Yes	159	63.3	
	No	92	36.7	
Take medications with supervision?				
	Yes	90	35.9	
	No	161	64.1	
Current Smoking status				
	Yes	27	10.8	
	No	224	89.2	
History of smoking				
	Yes	44	17.5	
	No	207	82.5	
Healthy diet				
	Yes	72	28.7	
	No	179	71.3	
On antihypertensive medication?				
	Yes	26	10.4	
	No	225	89.6	
On antidiabetic medication?				
	Yes	34	13.5	
	No	217	86.5	
On statins?				
	Yes	12	4.8	
	No	239	95.2	

Abbreviation: DM (Diabetes Mellitus) HTN (Hypertension), SD (standard deviation).

*Number of psychiatric inpatient admissions; extracted from clinical records.

The overall prevalence of MetS was 29.9% based on the Modified ATP III-A criteria, 30.3% according to the International Diabetes Federation (IDF) criteria, and 33.1% based on the Joint Statement criteria. For more details, see Table 3.

**Table 3:** Prevalence rate of metabolic syndrome among Omani adults with mental illnesses.

Category	*N*	%
Metabolic Syndrome ATP-III		
Yes	75	29.9
No	176	70.1
Metabolic Syndrome IDF		
Yes	76	30.3
No	175	69.7
Metabolic Syndrome Joint statement IDF		
Yes	83	33.1
No	168	66.9

A binary logistic regression analysis was conducted using a model-building approach to describe the relationship between Metabolic syndrome (dependent dichotomous variable) and the remaining independent variables. All independent variables significant at the bivariate level were included in the model. The final model for predictive factors of MetS showed Negelkerke R² = 0.529, *p* < 0.001. was with Negelkerke R^2^ = 0.529, *P* < 0.001. Additionally, the overall success prediction rate of the model was 81.8%.

After adjusting for other variables, greater waist circumference (OR = 1.063, 95% CI: 1.034–1.092, *p* < 0.001) and higher triglycerides (OR = 2.433, 95% CI: 1.401–4.219, *p* = 0.002) were associated with increased odds of MetS, whereas antidiabetic medication use (OR = 0.172, 95% CI: 0.040–0.732, *p* = 0.017) and higher physical activity (OR = 0.974, 95% CI: 0.949–0.997, *p* = 0.031) were associated with lower odds. Because waist circumference and triglycerides are integral diagnostic components of MetS, their associations were expected and interpreted as confirmation of definitional consistency rather than independent predictors. In contrast, the associations for antidiabetic medication use and physical activity reflect correlates beyond the diagnostic criteria. Other predictors, including educational level, age, number of medications, and comorbid chronic illness, were not statistically significant in this model. For further details, see Table 4.

**Table 4:** Associated factors of metabolic syndrome among Omani adults with mental illnesses.

Variables	B	S.E.	Wald	*P*	Exp(B)	95% C.I.for EXP(B)	
						Lower	Upper
Educational level [< Secondary Vs > secondary]	− 0.336	0.386	0.756	0.385	0.715	0.335	1.524
On antihypertensive? [Yes Vs No]	− 0.916	0.821	1.245	0.265	0.4	0.080	2.000
On antidiabetic? [Yes Vs No]	-1.763	0.740	5.677	**0.017**	0.172	0.040	0.732
On statins? [Yes Vs No]	-1.768	1.127	2.459	0.117	0.171	0.019	1.555
Age	0.013	0.021	0.370	0.543	1.013	0.973	1.055
Total number of medications	0.306	0.252	1.472	0.225	1.358	0.829	2.222
Total number of chronic illnesses	− 0.698	0.798	0.765	0.382	0.498	0.104	2.374
Godin Activity Level	− 0.027	0.012	4.669	**0.031**	0.974	0.949	0.997
Waist circumference size	0.061	0.014	19.578	**< 0.01**	1.063	1.034	1.092
Triglyceride blood level	0.889	0.282	9.962	**0.002**	2.433	1.401	4.219
Number of previous admissions	0.166	0.094	3.124	0.077	1.181	0.982	1.419
Diagnosed with a non-mental chronic illness? [Yes Vs No]	− 0.695	0.858	0.655	0.418	0.499	0.093	2.688
Constant	-3.639	2.659	1.872	0.171	0.026		

Abbreviation: Dependent Variable – Metabolic syndrome.

**ORs for continuous predictors reflect one-unit increases (waist circumference in cm; triglycerides in mmol/L; Godin score in points). Waist circumference and triglycerides are diagnostic components of MetS; their associations were expected and interpreted as confirmation of definitional consistency.

## Discussion

This study found that the prevalence of metabolic syndrome was 29.9%, 30.3%, and 33.1%, based on the Modified Adult Treatment Panel III-A (ATP III-A) criteria, the International Diabetes Federation (IDF) criteria, and the joint statement of the IDF Task Force with other organizations criteria, respectively. The close agreement among these criteria indicates robustness of the prevalence estimates. To the best of our knowledge, this is the first study to examine the prevalence and predictors of metabolic syndrome among adults with mental illnesses in Oman. The study’s novelty is primarily regional: it provides the first Oman-based estimates across different diagnostic definitions and situates metabolic risk within the country’s tertiary psychiatric outpatient context.

The current study’s results align with those reported from Belgium. (32.5%)^24^, South Africa (32%)^25^, and Nepal (30%) based on the ATP III-A definition^26^. Nevertheless, the rate of metabolic syndrome among Omani adults with mental illnesses exceeded the reported prevalence in Singapore (26.9%) based on ATP III-A^27^ and Kuwait (23.2%), (24.9%) based on the Modified Adult Treatment Panel III-A (ATP III-A) criteria and the International Diabetes Federation (IDF) criteria, respectively^28^. On the other hand, the findings of the current study showed a lower prevalence of metabolic syndrome compared to the reported prevalence in Saudi Arabia. (41.2%)^29^, United Arab Emirates (48.1%)^30^, Egypt (38.1%)^31^, Qatar (35.4%)^32^, based on IDF criteria, and from Palestine (43.6%) based on ATP III definition^33^.

In Gulf Cooperation Council (GCC) countries, the prevalence of metabolic syndrome (MetS) in the general population is high, with an overall estimate of 40%^32^. This level appears to be approximately 10–15% points higher than estimates reported for many developed regions globally^34^. Urbanization and associated lifestyle changes may partly explain the higher prevalence observed among adults with mental illnesses in this study.

### Associated factors

Several significant associated factors were identified in the current study, including the use of antidiabetic medications, which were significantly associated with lower odds of metabolic syndrome. This finding underscores the crucial role of pharmacotherapy in managing MetS by targeting multiple components, including blood glucose, blood pressure, weight, inflammation, and plasma lipids^35^. The observation of glucose intolerance in untreated patients and their family members suggests a genetic predisposition to metabolic disturbances, emphasizing the need for early pharmacological intervention; our results further underscore that antidiabetic medication may help mitigate disease progression in these at-risk individuals^36^. Moreover, pharmacotherapy plays a crucial role in mitigating metabolic and cardiovascular risks by targeting individual syndrome components and preventing complications^37^.

Another study highlighting the importance of recognizing metabolic syndrome as a separate diagnosis in patients with mental illnesses found that antidiabetic medications reduced the odds of developing MetS by 32%^38^. Collectively, these findings suggest that antidiabetic medication not only manages hyperglycemia but also offers broader protective effects against MetS, supporting its use as a key predictive factor and therapeutic strategy.

Results also indicated that low activity levels were strongly associated to MetS, explaining the notable decrease in life expectancy among patients with mental illnesses. This is because reduced activity contributes to a decline in physical health, driven by low motivation for regular activities, ultimately leading to significant health issues^39,40^. In parallel with this, several studies have shown that MetS is associated with low physical activity and obesity^39,41–44^. Increasing activity levels in patients with mental illnesses is essential for reducing metabolic changes^45^. Integrating structured physical activity programs into mental health services and regularly screening for MetS risk factors are essential for minimizing its effects on individuals with mental health issues. A collaborative approach that involves mental health professionals, dietitians, and fitness specialists can promote healthier lifestyle choices and enhance patient engagement. Moreover, focused education and behavioral interventions should address obstacles such as low motivation to enhance commitment to physical activity. By adopting these strategies, we can reduce metabolic complications, thereby improving life expectancy and overall well-being in this group.

This study showed that waist circumference was positively correlated with MetS. However, as waist circumference is a diagnostic component of MetS across the criteria used in this study, its statistical association with MetS is expected and reflects definitional consistency rather than a novel independent predictor. This was consistent with many previous reports^24,28,46^. Waist circumference measures central obesity and acts as an indicator of excessive visceral fat accumulation^47,48^. Excess abdominal fat, especially visceral fat, is strongly associated with the development of MetS^20^. Additionally, the link between central obesity and insulin resistance shows a stronger correlation than that of lower-body obesity^49^. Several studies reported a high prevalence of increased waist circumference among individuals with mental illnesses^24,50^. This may be due to the dietary habits of individuals with mental illnesses, as they tend to consume processed food that contains excessive salt and lacks fruits and vegetables compared with the general population^51^. Furthermore, psychotropic medications induce weight gain, resulting in higher obesity rates among patients with mental illnesses^52^. Accordingly, we do not interpret the waist circumference finding as an independent predictive relationship; instead, we emphasize modifiable, non-definitional correlates identified in our model, like physical activity.

Rothberg, McEwen^53^ found that greater reductions in waist circumference resulting from weight loss are linked to more substantial improvements in various related factors MetS. Moreover, nutrition interventions have been proven to significantly influence weight, BMI, and waist circumference in individuals with serious mental illnesses^46^. Therefore, healthcare providers are strongly encouraged to incorporate nutritional interventions and weight management programs at earlier stages of treatment for individuals with mental illnesses.

In this study, triglyceride (TG) levels in the blood were a significant factor associated with MetS. However, TG is a diagnostic component of MetS in the applied criteria; therefore, its observed significance is expected and should not be construed as a novel independent association. In line with this definitional overlap, we present the TG association as confirmatory rather than independent. Most individuals with MetS tend to have elevated plasma triglyceride levels^54^. Elevated serum triglyceride levels are a common sign of dyslipidemia and often occur alongside other cardiovascular risk factors, such as central obesity and high blood pressure^55^. The link between high triglyceride blood levels and metabolic syndrome is not fully understood^56^. However, a hypothesis proposes that the buildup of triglycerides in skeletal muscles directly contributes to insulin resistance, which can lead to MetS^57^.

At the multivariate level, the current study revealed that individuals with comorbidities, such as hypertension, did not show an association with MetS when compared to patients without comorbidities. However, earlier research indicates that patients with mental illnesses have a higher prevalence of physical comorbidities compared to the general population^14,29,32,40^. The age of the study participants may account for this variance in results. The average age of the participants in this study was 38.4 years, with the majority being younger than 40 years. Therefore, further research in Oman is required to confirm or refute the findings of the current study.

### Recommendations

The prevalence of MetS are consistent with international evidence; hence, we recommend the following service-focused actions: embed a standard metabolic screening bundle (waist circumference, blood pressure, fasting plasma glucose, Lipid profile) at intake and periodic follow-up, with results recorded in the mental health record. Furthermore, implement structured physical-activity counselling using brief tools (e.g., GSLTPAQ) with referral pathways to dietetics/weight-management. Moreover, deliver in-service training to ensure measurement fidelity and consistent referral; and add a registry field for MetS status to enable audit and quality improvement. Last but not least and to evaluate effectiveness without implying causality, future research should include pragmatic service evaluations and longitudinal or experimental designs with diagnosis-specific stratification. It should also be noted that the present study analyzed all participants with mental illnesses as a single group. While this approach provided a comprehensive overview of metabolic risk among adults with psychiatric conditions, it did not allow for differentiation by specific diagnoses such as schizophrenia, bipolar disorder, or major depressive disorder. Future studies with larger, diagnosis-balanced samples should perform stratified analyses to identify disorder-specific metabolic risk profiles and enable more tailored screening and intervention strategies.

### Limitations

One of the design’s inherited limitations of the current study is that, due to its cross-sectional design, temporal relationships between exposure factors and the development of metabolic syndrome cannot be established. Second, the use of a convenience sampling method may limit external validity, as participants were recruited from two tertiary psychiatric hospitals, which may not represent adults with mental illnesses across Oman. Third, although objective measurements were used for metabolic variables, some data, such as physical activity, were self-reported, which may introduce recall and social desirability bias. Fourth, incomplete data on the type, dose, and duration of psychotropic medications limited the ability to assess medication-related metabolic risk, potentially resulting in residual confounding. Specifically, antipsychotic class (typical vs. second-generation), agent, dose/formulation, and duration were not consistently available in outpatient records; only “current psychotropic use” (inclusion requirement) was uniform. As second-generation antipsychotics are strongly associated with metabolic disturbances, lack of exposure detail may confound observed associations. Future studies should prospectively capture antipsychotic exposure (agent, class, dose, duration) and link to dispensing data to enable valid adjustment. Fifth, all psychiatric conditions were analyzed as a single group, without differentiation by diagnostic subtype; future studies should stratify by diagnosis to identify disorder-specific metabolic patterns. Sixth, the study did not include dietary intake or sleep quality measures, both of which are known metabolic risk modifiers. Finally, the relatively young sample (mean age 38 years) may have led to an underestimation of age-related comorbidities. Additionally, some variables included in the regression model (e.g., waist circumference, triglycerides, and blood pressure) are definitional elements of MetS; although included to illustrate their relative contributions, this approach may introduce circularity, and these findings were interpreted with caution. Despite these limitations, this study provides the first national evidence on the coexistence of mental illness and metabolic syndrome in Oman. It lays the groundwork for future longitudinal and interventional studies.

## Conclusion

This study provides the first Oman-based estimates of the prevalence of metabolic syndrome among adults with mental illnesses and identifies factors associated with the syndrome. In multivariable analysis, larger waist circumference and higher triglyceride levels were associated with greater odds of metabolic syndrome. In contrast, higher physical activity and the use of antidiabetic medication were associated with lower odds. These findings are associative and do not establish directionality or causation. Because waist circumference and triglycerides are definitional components, their associations reflect diagnostic consistency rather than independent risk effects. The results highlight the practical value of incorporating routine metabolic screening and structured lifestyle support programs into psychiatric services in Oman. These should be prioritized for future service development and evaluated through longitudinal or experimental studies.

## Data Availability

Data cannot be shared publicly because of ethical restrictions. Data are available from the Ethics Committee (adpsr\_nurs@squ.edu.om) for researchers who meet the criteria for access to confidential data.

## References

[1] American Psychiatric Association. Diagnostic and Statistical Manual of Mental Disorders (5th ed.). American Psychiatric Publishing,Washington, DC (2013).10.1176/appi.books.9780890425596

[2] WHO. Mental health. [cited 2022. Available from: https://www.who.int/health-topics/mental-health#tab=tab_1 (2022).

[3] Dattani, S., Ritchie, H. & Roser, M. *Mental Health* (Our world in data, 2021).

[4] Murray & Lopez. The global burden of disease: a comprehensive assessment of mortality and disability from diseases, injuries, and risk factors in 1990 and projected to 2020. (2020).

[5] Fiorillo, A. & Sartorius, N. Mortality gap and physical comorbidity of people with severe mental disorders: the public health scandal. *Ann. Gen. Psychiatry*. **20**, 1–5 (2021). 34903254 10.1186/s12991-021-00374-y PMC8670051

[6] Afzal, M. et al. Prevalence of overweight and obesity in people with severe mental illness: systematic review and meta-analysis. *Front. Endocrinol.* **12**, 769309 (2021). 10.3389/fendo.2021.769309 PMC8656226 34899604

[7] Gouveia, É. R. et al. Predictors of metabolic syndrome in adults and older adults from Amazonas, Brazil. *Int. J. Environ. Res. Public Health*. **18**10.3390/ijerph18031303 (2021). 10.3390/ijerph18031303 PMC7908119 33535582

[8] Vancampfort, D. et al. Risk of metabolic syndrome and its components in people with schizophrenia and related psychotic disorders, bipolar disorder and major depressive disorder: a systematic review and meta-analysis. *World Psychiatry*. **14** (3), 339–347 (2015). 26407790 10.1002/wps.20252 PMC4592657

[9] Chan, J. K. N. et al. Life expectancy and years of potential life lost in people with mental disorders: a systematic review and meta-analysis. *EClinicalMedicine*. **65**. 102294 10.1016/j.eclinm.2023.102294(2023). 10.1016/j.eclinm.2023.102294 PMC10641487 37965432

[10] De Hert, M. et al. Physical illness in patients with severe mental disorders. I. Prevalence, impact of medications and disparities in health care. *World Psychiatry*. **10** (1), 52 (2011). 21379357 10.1002/j.2051-5545.2011.tb00014.x PMC3048500

[11] Schuch, F. et al. Physical activity and sedentary behavior in people with major depressive disorder: a systematic review and meta-analysis. *J. Affect. Disord.* **210**, 139–150 (2017). 28033521 10.1016/j.jad.2016.10.050

[12] Correll, C. U. et al. Prevalence, incidence and mortality from cardiovascular disease in patients with pooled and specific severe mental illness: A large-scale meta-analysis of 3,211,768 patients and 113,383,368 controls: Correction. (2018). 10.1002/wps.20420 PMC5428179 28498599

[13] Kang, M., Joo, M. & Hong, H. Eating Speed, physical Activity, and cardiorespiratory fitness are independent predictors of metabolic syndrome in Korean university students. *Nutrients* **13** (7), 2420 (2021). 34371929 10.3390/nu13072420 PMC8308714

[14] Firth, J. et al. The lancet psychiatry commission: a blueprint for protecting physical health in people with mental illness. *Lancet Psychiatry*. **6** (8), 675–712 (2019). 31324560 10.1016/S2215-0366(19)30132-4

[15] Pereira, L., Budovich, A. & Claudio-Saez, M. Monitoring of metabolic adverse effects associated with atypical antipsychotics use in an outpatient psychiatric clinic. *J. Pharm. Pract.* **20** (18), 179–179 (2018). 10.1177/0897190017752712 29334810

[16] Stanley, S. H. et al. Kimberley Indigenous mental health: an examination of metabolic syndrome risk factors. *Aust. J. Rural Health*. **24** (5), 300–305 (2016). 26689845 10.1111/ajr.12270

[17] Musa, M. Mental health in the middle east: historical perspectives, current challenges, and future implications. *Saudi J. Humanit. Soc. Sci.* **9** (5), 138–148 (2024).

[18] Musa, M. E. Pre-settlement and Post-settlement Stressors and Mental Illness Among Immigrants from War-torn Countries in the Middle East: A Scoping Review. (2024).

[19] Aboul Enein, B. H., Bernstein, J. & Neary, A. Dietary transition and obesity in selected Arabic-speaking countries: a review of the current evidence. *EMHJ-Eastern Mediterranean Health J.* **22** (10), 763–770 (2016). 10.26719/2016.22.10.763 28134430

[20] Parley, C. A. & Johnson, M. I. Abdominal Obesity and Metabolic Syndrome: Exercise as Medicine (BMC Sports Science Medicine Rehabilitation. 2018). 10.1186/s13102-018-0097-1 PMC5935926 29755739

[21] Alberti et al. Harmonizing the metabolic syndrome. *Circulation* **120** (16), 1640–1645 (2009). 19805654 10.1161/CIRCULATIONAHA.109.192644

[22] Amireault, S. & Godin, G. The Godin-Shephard leisure-time physical activity questionnaire: validity evidence supporting its use for classifying healthy adults into active and insufficiently active categories. *Percept. Mot. Skills*. **120** (2), 604–622 (2015). 25799030 10.2466/03.27.PMS.120v19x7

[23] Asiri, F. et al. Cross-Cultural adaptation of the Godin-Shephard Leisure-Time physical activity questionnaire for Arabic population and testing its psychometric properties. *Med. Sci. Monitor: Int. Med. J. Experimental Clin. Res.* **28**, e937245–e937241 (2022). 10.12659/MSM.937245 PMC9426207 36002999

[24] Mitchell, A. J. et al. Prevalence of metabolic syndrome and metabolic abnormalities in schizophrenia and related disorders—a systematic review and meta-analysis. *Schizophr. Bull.* **39** (2), 306–318 (2013). 22207632 10.1093/schbul/sbr148 PMC3576174

[25] Maaroganye, K. et al. The prevalence of metabolic syndrome and its associated factors in long-term patients in a specialist psychiatric hospital in South Africa. *Afr. J. Psychiatry (Johannesbg)*. **16**(6). 10.4314/ajpsy.v16i6.53 (2013). 10.4314/ajpsy.v16i6.53 24173635

[26] Subedi, A., Lamichhane, R. P. & Khattri, J. B. Study of metabolic syndrome in schizophrenic patients treated with antipsychotics. *J. Chitwan Med. Coll.* **10** (4), 29–33 (2020).

[27] Seow, L. S. E. et al. Metabolic syndrome and cardiovascular risk among institutionalized patients with schizophrenia receiving long term tertiary care. *Compr. Psychiatr.* **74**, 196–203 (2017). 10.1016/j.comppsych.2017.01.017 28214752

[28] Roshdy, R. et al. Prevalence of the metabolic syndrome by five definitions, and its associations among consecutive admissions to an acute admission psychiatric care facility in an Arab setting. *Middle East. J. Psychiatry Alzheimers*. **2**(2). 3–9 (2011).

[29] Alosaimi, F. D. et al. Prevalence of metabolic syndrome and its components among patients with various psychiatric diagnoses and treatments: A cross-sectional study. *Gen. Hosp. Psychiatry*. **45**, 62–69 (2017). 28274341 10.1016/j.genhosppsych.2016.12.007

[30] Shahda, M. & El-Sayed, A. Study of the prevalence of metabolic syndrome among psychiatric patients and its correlation with diagnosis and medications. *Egypt. J. Psychiatry (Print)*. **31**, n2 (2010).

[31] Hatata, H. et al. Risk factors of metabolic syndrome among Egyptian patients with schizophrenia. *Curr. Psychiatry*. **16**, 85–95 (2009).

[32] Hammoudeh et al. The prevalence of metabolic syndrome in patients receiving antipsychotics in qatar: a cross sectional comparative study. *BMC Psychiatry*. **18** (1), 81 (2018). 29587717 10.1186/s12888-018-1662-6 PMC5870932

[33] Sweileh, W. M. et al. Prevalence of metabolic syndrome among patients with schizophrenia in Palestine. *BMC Psychiatry*. **12** (1), 1–8 (2012). 23270504 10.1186/1471-244X-12-235 PMC3543707

[34] Mabry, R. et al. Gender differences in prevalence of the metabolic syndrome in Gulf Cooperation Council countries: a systematic review. *Diabet. Med.* **27** (5), 593–597 (2010). 20536958 10.1111/j.1464-5491.2010.02998.x

[35] Stanciu, S. et al. Links between metabolic syndrome and hypertension: the relationship with the current antidiabetic drugs. *Metabolites* **13** (1), 87 (2023). 36677012 10.3390/metabo13010087 PMC9863091

[36] Lopuszanska, U. J. et al. Mental illness and metabolic syndrome-a literature review. *Ann. Agric. Environ. Med.*, **21**(4), 815–82110.5604/12321966.1129939 (2014). 10.5604/12321966.1129939 25528926

[37] Rustamovna, M. R. Metabolic syndrome and its pharmacotherapy at the modern level. *Int. J. Med. Sci. Clin. Res.* **4** (05), 36–41 (2024).

[38] Noam, K. et al. Predictors of metabolic syndrome (MetS) and the benefits of using the MetS diagnosis for people with serious and persistent mental illness. *J. Psychiatr. Res.* **187**, 108–115 (2025). 40359803 10.1016/j.jpsychires.2025.04.023

[39] Erginer, D. K. & Günüşen, N. P. Determination of physical health status and healthy lifestyle behaviors of individuals with mental illness. *Perspect. Psychiatr. Care.* **54** (3), 371–379 (2018). 29473170 10.1111/ppc.12261

[40] Reininghaus, B. et al. Physical health in individuals with psychiatric disorders in Austria. *J. Affect. Disord.* **257**, 38–44 (2019). 31299403 10.1016/j.jad.2019.06.045

[41] De Hert, M., Detraux, J. & Vancampfort, D. *The Intriguing Relationship between Coronary Heart Disease and Mental Disorders* (Dialogues in clinical neuroscience, 2022). 10.31887/DCNS.2018.20.1/mdehert PMC6016051 29946209

[42] Severi, E. et al. Assessment of cardiovascular risk in an Italian psychiatric outpatient sample: A chart review of patients treated with second-generation antipsychotics. *Int. J. Ment. Health Nurs.* **27** (3), 1002–1008 (2018). 29197134 10.1111/inm.12407

[43] Weiser, P. et al. European network for promoting the physical health of residents in psychiatric and social care facilities (HELPS): background, aims and methods. *BMC Public. Health*. **9** (1), 1–9 (2009). 19715560 10.1186/1471-2458-9-315 PMC2741451

[44] Sisson, S. B. et al. Screen time, physical activity, and overweight in US youth: National survey of children’s health 2003. *J. Adolesc. Health*. **47** (3), 309–311 (2010). 20708572 10.1016/j.jadohealth.2010.02.016

[45] Stubbs, B. et al. How sedentary are people with psychosis? A systematic review and meta-analysis. *Schizophr. Res.* **171** (1–3), 103–109 (2016). 26805414 10.1016/j.schres.2016.01.034

[46] Teasdale, S. B. et al. Solving a weighty problem: systematic review and meta-analysis of nutrition interventions in severe mental illness. *Br. J. Psychiatry*. **210** (2), 110–118 (2017). 27810893 10.1192/bjp.bp.115.177139

[47] Wakabayashi, I. Necessity of both waist circumference and waist-to-height ratio for better evaluation of central obesity. *Metab. Syndr. Relat. Disord.* **11** (3), 189–194 (2013). 23451815 10.1089/met.2012.0131

[48] Deedwania, P. & Gupta, R. Management issues in the metabolic syndrome. *JAPI* **54**, 797–810 (2006). 17214277

[49] Westphal, S. A. Obesity, abdominal obesity, and insulin resistance. *Clin. Cornerstone*. **9** (1), 23–31 (2008). 19046737 10.1016/s1098-3597(08)60025-3

[50] De Hert, M. et al. Metabolic and cardiovascular adverse effects associated with antipsychotic drugs. *Nat. Reviews Endocrinol.* **8** (2), 114–126 (2012). 10.1038/nrendo.2011.156 22009159

[51] Dipasquale, S. et al. The dietary pattern of patients with schizophrenia: a systematic review. *J. Psychiatr. Res.* **47** (2), 197–207 (2013). 23153955 10.1016/j.jpsychires.2012.10.005

[52] Correll, C. U. et al. Antipsychotic combinations vs monotherapy in schizophrenia: a meta-analysis of randomized controlled trials. *Schizophr. Bull.* **35** (2), 443–457 (2009). 18417466 10.1093/schbul/sbn018 PMC2659301

[53] Rothberg, A. E. et al. Impact of weight loss on waist circumference and the components of the metabolic syndrome. *BMJ Open. Diabetes Res. Care*. **5** (1), e000341 (2017). 28316795 10.1136/bmjdrc-2016-000341 PMC5337678

[54] Grundy, M. M. L. et al. Re-evaluation of the mechanisms of dietary fibre and implications for macronutrient bioaccessibility, digestion and postprandial metabolism. *Br. J. Nutr.* **116** (5), 816–833 (2016). 27385119 10.1017/S0007114516002610 PMC4983777

[55] Lin, L. Y. et al. The effects of indoor particle exposure on blood pressure and heart rate among young adults: an air filtration-based intervention study. *Atmos. Environ.* **45** (31), 5540–5544 (2011).

[56] Cornier, M. A. et al. The metabolic syndrome. *Endocr. Rev.* **29** (7), 777–822 (2008). 18971485 10.1210/er.2008-0024 PMC5393149

[57] McGarry, J. D. Banting lecture 2001: dysregulation of fatty acid metabolism in the etiology of type 2 diabetes. *Diabetes* **51** (1), 7–18 (2002). 11756317 10.2337/diabetes.51.1.7

